# Outpatient antibiotic prescribing for common infections via telemedicine versus face-to-face visits: Systematic literature review and meta-analysis

**DOI:** 10.1017/ash.2021.179

**Published:** 2021-08-31

**Authors:** Hiroyuki Suzuki, Alexandre R. Marra, Shinya Hasegawa, Daniel J. Livorsi, Michihiko Goto, Eli N. Perencevich, Michael E. Ohl, Jennifer DeBerg, Marin L. Schweizer

**Affiliations:** 1 Center for Access & Delivery Research & Evaluation (CADRE), Iowa City Veterans Affairs Health Care System, Iowa City, Iowa, United States; 2 Department of Internal Medicine, University of Iowa Carver College of Medicine, Iowa City, Iowa, United States; 3 Instituto Israelita de Ensino e Pesquisa Albert Einstein, Hospital Israelita Albert Einstein, São Paulo, Brazil; 4 Division of Infectious Diseases, Tokyo Metropolitan Tama Medical Center, Tokyo, Japan; 5 Hardin Library for the Health Sciences, University of Iowa Libraries, Iowa City, Iowa, United States

## Abstract

**Objective:**

To evaluate the frequency of antibiotic prescribing for common infections via telemedicine compared to face-to-face visits.

**Design:**

Systematic literature review and meta-analysis.

**Methods:**

We searched PubMed, CINAHL, Embase (Elsevier platform) and Cochrane CENTRAL to identify studies comparing frequency of antibiotic prescribing via telemedicine and face-to-face visits without restrictions by publish dates or language used. We conducted meta-analyses of 5 infections: sinusitis, pharyngitis, otitis media, upper respiratory infection (URI) and urinary tract infection (UTI). Random-effect models were used to obtain pooled odds ratios (ORs). Heterogeneity was evaluated with I^2^ estimation and the Cochran Q statistic test.

**Results:**

Among 3,106 studies screened, 23 studies (1 randomized control study, 22 observational studies) were included in the systematic literature review. Most of the studies (21 of 23) were conducted in the United States. Studies were substantially heterogenous, but stratified analyses revealed that providers prescribed antibiotics more frequently via telemedicine for otitis media (pooled odds ratio [OR], 1.26; 95% confidence interval [CI], 1.04–1.52; I^2^ = 31%) and pharyngitis (pooled OR, 1.16; 95% CI, 1.01–1.33; I^2^ = 0%). We detected no significant difference in the frequencies of antibiotic prescribing for sinusitis (pooled OR, 0.86; 95% CI, 0.70–1.06; I^2^ = 91%), URI (pooled OR, 1.18; 95% CI, 0.59–2.39; I^2^ = 100%), or UTI (pooled OR, 2.57; 95% CI, 0.88–7.46; I^2^ = 91%).

**Conclusions:**

Telemedicine visits for otitis media and pharyngitis were associated with higher rates of antibiotic prescribing. The interpretation of these findings requires caution due to substantial heterogeneity among available studies. Large-scale, well-designed studies with comprehensive assessment of antibiotic prescribing for common outpatient infections comparing telemedicine and face-to-face visits are needed to validate our findings.

In the United States, ∼60% of antimicrobial expenditures are associated with the outpatient setting,^1^ and at least 30% of outpatient antibiotic prescriptions are potentially unnecessary.^2^ Thus, targets of antimicrobial stewardship programs (ASPs) should also focus on outpatient antibiotic prescribing.

Telemedicine is the provision of health care remotely using various telecommunication tools such as phone visits or mobile devices with or without a video connection.^3^ Before the coronavirus disease 2019 (COVID-19) pandemic, telemedicine was relatively uncommon in the United States, although its use was gradually increasing.^4^ However, since the beginning of the COVID-19 pandemic, telemedicine use has rapidly increased.^5,6^ Although telemedicine could provide an effective and safer alternative to face-to-face visits in many clinical contexts during the pandemic, there is not enough evidence of how provider antibiotic prescribing varies according to the mode of care delivery. Diagnostic uncertainty may increase for some infections because reliable physical examination and diagnostic tests are not always available via telemedicine. Providers may overprescribe antibiotics via telemedicine due to anxiety about missing bacterial infections. Furthermore, patients’ demand for antibiotics and providers’ perceptions of this demand may be different via telemedicine. Previous studies have suggested that telemedicine was associated with increased antibiotic prescribing.^7,8^ One systematic review conducted in early 2020 suggested an association between telemedicine and outpatient antibiotic prescribing in primary care settings.^9^ However, the study results were mixed and too heterogeneous to conduct a meta-analysis. Additionally, that study excluded ambulatory care settings other than primary care.

To address this knowledge gap, we conducted a systematic literature review and meta-analysis of the frequency of outpatient antibiotic prescribing via telemedicine versus face-to-face visits by including all visit settings not limiting to primary care settings to better describe variations in antibiotic prescribing according to the mode of care delivery (PROSPERO registration no. CRD42021228585).

## Methods

### Systematic literature review and search strategies

This systematic literature review and meta-analysis were conducted according to the Preferred Reporting Items for Systematic Reviews and Meta-analyses (PRISMA) statement^10^ and Meta-analysis of Observational Studies in Epidemiology (MOOSE) guidelines.^11^ Search strategies were developed with the assistance of a health sciences librarian with expertise in searching for systematic reviews in December 2020 and January 2021. The literature search included publications from database inception to January 15, 2021. Comprehensive strategies, including both index and keyword methods, were devised for the following databases: PubMed, CINAHL, Embase (Elsevier platform) and Cochrane CENTRAL. To maximize sensitivity, no pre-established database filters were used. The full PubMed search strategy (Supplementary Table 1 online) was adapted for the other databases. In addition to the database searches, references of 14 relevant papers were located using the Scopus database. Publications were included if they evaluated the frequency of antibiotic prescribing in outpatient settings via telemedicine. Studies were excluded if they did not have a control group (ie, face-to-face visits). Titles and abstracts of the studies identified by the initial literature search were screened (by H.S.) to assess inclusion criteria. The authors of 6 studies were contacted to provide additional information needed for meta-analysis. Among them, 1 author provided additional information, and that study was included in the meta-analysis.^12^

**Table 1.** Summary of Study Characteristics

First Author/Publication Year/Location	Setting	Study Design	Adjustment for Confounders	Study Period	Adult/ Children	Type of Telemedicine	Type of Face-to-Face Visit	Infectious Diagnosis for Antibiotics	Identification of Diagnosis	Comments	D&B Score
Bruxvoort, 2020, California^19^	Kaiser Permanente Southern California	Retrospective cohort study	None	10 y	Adult/ Children	Both synchronous and asynchronous	Clinic visit, emergency department	UTI	Administrative codes	Study investigated time trend of telemedicine and face-to-face visit over 10 y; includes both cystitis and pyelonephritis. UTI with antibiotic in telemedicine in female increased by 21.2% per year whereas that in office visits declined 2.8% per year.	19
Davis, 2018, Colorado^15^	UC Health System	Retrospective cohort study	None	12 mo	Adults	Synchronous video/phone visit	Urgent care	Sinusitis	Administrative codes	Age <18 years and >89 years were excluded.	17
Ewen, 2015, Delaware^20^	Christiana Care Health System	Retrospective cohort study	None	5 y	Adults/ Children	Synchronous phone visit	Clinic visit	Various conditions (UTI, sinusitis, URI, bronchitis, pharyngitis, genitourinary, cellulitis, gastroenteritis/intra-abdominal, pneumonia, Lyme disease, etc)	Chart review	Antibiotic prescribing was measured as rate/100 patient years. Antibiotic prescribing increased from 2.2 to 4.2 (telephone), and from 21.4 to 26.1 (face-to-face visit) during the study.	20
Gordon, 2017 United States^30^	HealthCore Integrated Research Database	Cross-sectional study	Face-to-face visits were matched to virtual visits with 3:1 ratio on acute condition, quarter/year of index date, state/region of the residence and age group.	1 y 5 mo	Adults/ Children	Both synchronous and asynchronous	Clinic visit, urgent care, emergency department, retail clinic	Sinusitis, pharyngitis, bronchitis, conjunctivitis, UTI, URI	Administrative codes	Follow-up care within 3 weeks in telemedicine (28.1%) was similar to PCP (28.1%) and retail clinic (28.6%), slightly more than urgent care (25.6%) and less than emergency department (34.2%). Lab usage in telemedicine (12.6%) was significantly less than all modes of face-to-face visits (36.8% in retail clinic, 39.0% in urgent care, 53.2% in emergency department and 37.4% with PCP).	21
Halpren-Ruder, 2019, Pennsylvania^23^	Thomas Jefferson University Hospital	Retrospective cohort study	Telemedicine visits were matched to face-to-face visits by visit dates	12 mo	Adults/ Children	Synchronous video visit	Urgent care, emergency department	Sinusitis	Chart review		19
Hersh, 2019, Utah^25^	Intermountain Healthcare	Retrospective cohort study	None	12 mo	Children	Synchronous video visit	Urgent care	Sinusitis, URI, pharyngitis, OM	Unspecified	Letter to editor	13
Huibers, 2014, Central Denmark^24^	Primary care network of central Denmark Region	Retrospective cohort study	None	12 mo	Adults/ Children	Synchronous phone visit	Clinic visit, home visit	Various conditions	Administrative codes	Antibiotic was prescribed more commonly in clinic (26.1%) than telephone visit (10.7%) or home visit (10.9%)	17
Johnson, 2019, Michigan^18^	Mercy Health Physician Partners primary care network	Retrospective cohort study	None	6 mo	Adult	Asynchronous text or internet visit	Clinic visit	Sinusitis	Administrative codes	Telemedicine used Zipnosis —antibiotic was chosen with drop-down menu. Outpatient antimicrobial stewardship program provided annual education. Antibiotic selection was similar between 2 groups. Of 25 patients who self-requested antibiotics, 100% of patients in the office visit and 63.2% in telemedicine were prescribed antibiotics (*P* = .08) More revisits occurred within 24 h in telemedicine (8% vs 1.7%) but no difference within 7 d (14.9% vs 16.6%).	23
Johnson, 2020, Michigan^14^	Mercy Health Physician Partners primary care network	Retrospective cohort study	None	12 mo	Adult	Asynchronous text or internet visit	Clinic visit	UTI	Administrative codes	Telemedicine used Zipnosis —antibiotic was chosen with a drop-down menu. Outpatient antimicrobial stewardship program provide annual education. Urinalysis (0% vs 97.1%) and urine culture (0% vs 73.1%) were less likely to be ordered during telemedicine. Office visit was associated with more revisits within 7 days in multivariate logistic regression analysis.	23
Lovell, 2019, Utah^31^	Intermountain Healthcare	Cross-sectional study	Telemedicine was randomly matched to face-to-face visits up to 3 claims based on age, primary diagnosis category, year, and quarter	12 mo	Adult/ Children	Synchronous video visit	Clinic visit, urgent care, emergency department	Sinusitis, URI, UTI, pneumonia, OM, bronchitis, conjunctivitis, cough, dermatitis/eczema, digestive system, ear pain, influenza/pneumonia	Administrative codes	Those aged >65 y were excluded. Follow-up care within 3 weeks in telemedicine (35.3%) was similar to PCP (35.7%) and urgent care (35.6%), and less than emergency department (73.0%) Lab usage in telemedicine (9.0%) was significantly less than all modes of face-to-face visits (27.5% in urgent care, 11.5% in emergency department and 25.7% with PCP).	21
McKinstry, 2002, West Lothian, UK^33^	Lothian Primary Care Research Network	Randomized controlled trial	Not available	1 mo	Adult/ Children	Synchronous phone visit	Clinic visit	Unspecified	Not available	In telephone visit group, 34 of 194 refused telephone visit and converted to face-to-face visit. Antibiotic was prescribed in 19.3% (35 of 181) in telephone visit and 16.0% (30 of 187) in face-to-face visit (difference, −3.3% (−11.1% to 4.5%).	22
Mehrotra, 2013, Pennsylvania^7^	University of Pittsburgh Medical Center system	Retrospective cohort study	None	1 y 4 mo	Unknown	Asynchronous text or internet visit	Clinic visit	Sinusitis, UTI	Administrative codes	For UTI, urine culture was ordered more commonly in office visits (31%) compared to telemedicine (7%) For sinusitis, sinus radiograph or computed tomography scan was rarely ordered (0% in telemedicine, 0.3% for office visits).	16
Miller, 2020 Massachusetts^32^	Partners Healthcare system	Case control study	None	3 mo	Adults	Unspecified	Clinic visit	Sinusitis	Administrative codes	Telemedicine group were recruited in 2020 during COVID-19 pandemic and face-to-face group were recruited in 2019 before the pandemic.	17
Murray, 2020, Minnesota^26^	Mayo Clinic	Retrospective cohort study	None	10 mo	Adults	Synchronous phone visit and asynchronous text or internet visit	Clinic visit, Retail clinic	UTI	Administrative codes, chart review	Those aged <18 y and >65 y were excluded. Urinalysis was less commonly ordered in eVisits (0%) and phone calls (5%) than in face-to-face visits (93%). Urine culture was less commonly ordered in eVisits (0%) and phone call (7%) than in face-to-face visits (21%). Similar follow-up rates among the 3 groups.	18
Norden, 2020, California^16^	Stanford’s ClickWell Care	Retrospective cohort study	None	2 y 1 mo	Adults	Synchronous video or phone visit	Urgent care	URI, OM, pharyngitis	Administrative codes	Patients with health risk assessment (HRA) score ≥5 were excluded. No difference in lab orders and imaging. Repeat visits within 1 d (40% vs 21%) and 3 d (53% vs 28%) were more common in telemedicine for pharyngitis.	17
Penza, 2020, Minnesota^28^	Mayo Clinic	Retrospective cohort study	None	12 mo	Children	Synchronous phone visit and asynchronous text or internet visit	Clinic visit, Retail clinic	Conjunctivitis	Administrative codes, chart review	Antibiotic was given more commonly via phone calls (41.6%) than in eVisits (25.7%) and face-to-face visits (19.8%). Healthcare workers recommended follow-up more commonly via phone calls (38.6%) than eVisits (29.7%), and far less commonly in face-to-face visits (1%).	19
Penza, 2020, Minnesota^27^	Mayo Clinic	Retrospective cohort study	None	12 mo	Adults	Synchronous phone visit and Asynchronous text/internet visit	Retail clinic	Sinusitis	Administrative codes, chart review	Healthcare workers recommended follow-up more commonly via phone calls (26%) than eVisits (3.3%) or face-to-face visits (0.7%). Of those who received antibiotics, >93% of patients received a guideline-recommended antibiotic, with no difference between groups.	18
Ray, 2019, United States^8^	Claim data from a large national insurer	Retrospective cohort study	Age, sex, chronic medical complexity, state, rural/urban, high-deductable health plan status and diagnosis category were matched.	2 y	Children	Synchronous video or phone visit	Clinic visit, urgent care	Sinusitis, URI, OM, streptococcal pharyngitis	Administrative codes	For streptococcal pharyngitis, streptococcal testing was offered less in telemedicine (4%) than with PCP (68%) or in urgent care (75%). For streptococcal pharyngitis, follow-up visits within 2 days were more common in telemedicine (5%) than with PCPs (1%) or in urgent care (2%).	20
Schmidt,2017, North Carolina^29^	Carolinas Healthcare System	Retrospective cohort study	None	3 y 2 mo	Unknown	Synchronous virtual visits and asynchronous text or internet visit	Clinic visit	Sinusitis, URI, OM, bronchitis	Unspecified	Conference abstract	16
Shi, 2018, United States^21^	Claim data from a large national insurer	Retrospective cohort study	Telemedicine was matched to face-to-face visits on age category, sex, chronic conditions, state, urbanicity of ZIP code, high-deductible health plan status, and diagnosis category.	2 y	Adult	Synchronous video or phone visit	Clinic visit, urgent care	Sinusitis, URI, OM, Streptococcus pharyngitis, bronchitis/bronchiolitis	Administrative codes	For streptococcal pharyngitis, a streptococcal testing was offered less in telemedicine (1%) compared to PCP (67%) or urgent care (78%). For streptococcal pharyngitis, follow-up visits within 21 d were more common in telemedicine (10%) than with PCP (6%) or urgent care (7%).	19
Tan, 2016, Nevada^12^	Southwest Medical	Retrospective cohort study	None	9 mo	Adults	Synchronous video visit	Urgent care	Viral URI	Administrative codes	Upper respiratory tract infection, common cold, sinusitis, bronchitis, pharyngitis, cough, and nasal congestion were included as “viral URI.” Those aged <18 y and >65 y were excluded. Telemedicine was an independent factor associated with revisiting within 2 weeks.	20
Uscher-Pines, 2015, California^22^	California Public Employee’s Retirement System	Retrospective cohort study	Antibiotic prescribing was assessed with multivariate models, adjusting for sex, age, chronic illness, site of care, and ARI diagnosis.	1 y 7 mo	Adults	Synchronous video or phone visit	Clinic visit	Sinusitis, URI, OM, pharyngitis, bronchitis, influenza	Administrative codes	Telemedicine with Teladoc	17
Yao, 2019, New York^17^	Weill Cornell Medical Center	Retrospective cohort study	Telemedicine visit was matched to in-person visit by diagnosis, treatment hospital and Emergency Severity Index level. Also adjusted for age and sex.	1 y 3 mo	Adults	Synchronous video visit	Emergency department	ARI (influenza, bronchitis, lung infection, URI/nasopharyngitis, sinusitis and OM)	Administrative codes	If the patient was determined to have a low-acuity complaint that was unlikely to require significant emergency department resources, a telemedicine visit was offered.	18

Note. UTI, urinary tract infection; URI, upper respiratory infection; OM, otitis media; ARI, acute respiratory infection; D&B score, Downs and Black score; PCP, primary care physician.

### Data abstraction and quality assessment

Of 3 independent reviewers (H.S., A.R.M., and S.H.), 2 abstracted data for each article using a standardized abstraction form. The reviewers abstracted data on publication year, study location, study setting, study design, study period, inclusion of adults and/or children, type of telemedicine, type of face-to-face visits, infectious diagnoses for which antibiotics were indicated, a definition of guideline-concordant antibiotic management, and an assessment of the potential risk of bias. Our primary outcome was the frequency of antibiotic prescribing via telemedicine and face-to-face visits, defined as the proportion of total visits in which an antibiotic was prescribed. As a secondary outcome, we evaluated guideline-concordant antibiotic management. We decided to conduct meta-analyses for individual diagnoses but not all diagnoses together.

The risk of bias was assessed by independent reviewers using the Downs and Black scale.^13^ All questions of the original Downs and Black scale were answered as intended except a categorical question that we changed to a dichotomous answer for convenience. The maximum score was 28 points. Studies that scored 18 points or more were considered high quality. For data abstraction and quality assessment, inconsistent assessments were resolved by discussion.

### Statistical analysis

To estimate the pooled odds ratio (OR) and 95% confidence interval (CI) for each infection, we used random-effects models with inverse variance weighting. We performed stratified analyses by the modes of telemedicine, location of face-to-face visits, adults or children, year of publication, and risk of bias according to the Downs and Black scale. Heterogeneity was evaluated with I^2^ estimation and the Cochran Q statistic test. We used the Cochrane Review Manager (Revman) version 5.4 (The Nordic Cochrane Centre, The Cochrane Collaboration. Copenhagen, 2014). Publication bias was assessed using funnel plots.

## Results

### Systematic literature review of antibiotic prescribing in telemedicine versus face-to-face visits

**Fig. 1.**
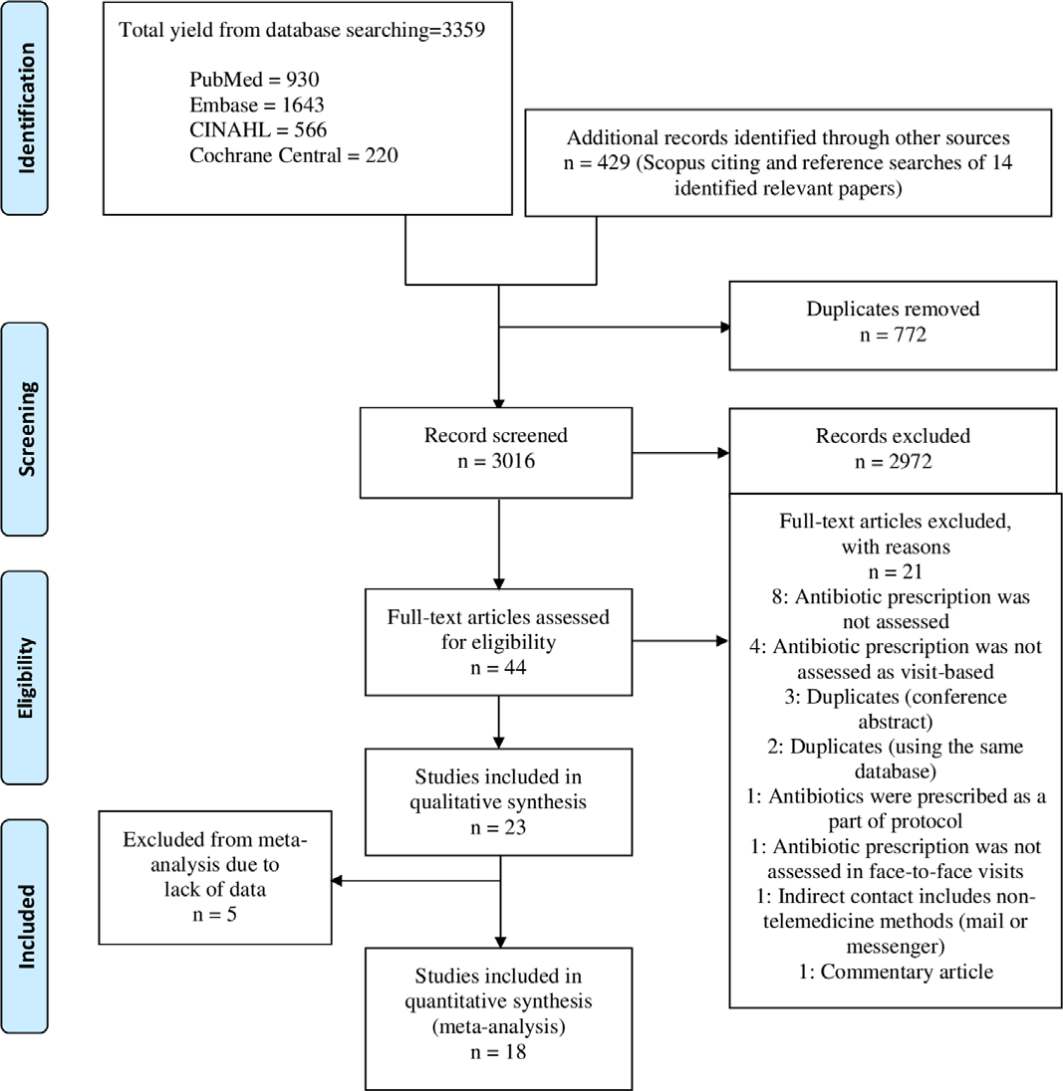
Flow diagram of literature search adapted from PRISMA flow chart.

Among 3,106 studies screened, 23 studies met the inclusion criteria and were included in the systematic literature review (Fig. 1). Of these 23 studies, 19 were retrospective cohort studies,^7,8,12,14–29^ 2 were cross-sectional studies,^30,31^ 1 was a case–control study,^32^ and 1 was a randomized controlled trial^33^ (Table 1). Of the 22 observational studies, 7 studies used matching between the exposed group (telemedicine) and the nonexposed group (face-to-face visits).^8,17,21–23,30,31^ Of 23 studies, 21 were conducted in the United States,^7,8,12,14–23,25–32^ 1 was conducted in Denmark,^24^ and 1 was conducted in the United Kingdom.^33^ Of the 21 studies conducted in the United States, 4 studies used a claim-based database^8,21,22,30^ and the others were conducted either in a single healthcare system or in a primary care network.

Of the 23 studies, 11 included only adults,^12,14–18,21,22,26,27,32^ 7 investigated both adults and children,^19,20,23,24,30,31,33^ and 3 involved only children.^8,25,28^ For telemedicine modalities, 17 studies evaluated synchronous video and/or phone visits,^8,12,15–17,20–29,31,33^ 7 evaluated asynchronous text or internet visits,^7,14,18,26–29^ and 3 neither specified nor separated those 2 modalities.^19,30,32^ Also, 16 studies evaluated clinic visits,^7,8,14,18–22,24,26–33^ 9 evaluated urgent care,^8,12,15,16,21,23,25,30,31^ 5 evaluated emergency departments,^17,19,23,30,31^ and 4 evaluated retail clinic visits.^26–28,30^

The most commonly evaluated indication was sinusitis, which was reported in 10 studies,^7,8,15,18,21–23,27,30,32^ followed by upper respiratory infection (URI), which was reported in 6 studies,^8,12,16,21,22,30^ urinary tract infection (UTI), which was reported in 5 studies,^7,14,19,26,30^ pharyngitis, which was reported in 5 studies,^8,16,21,22,30^ and otitis media, which was reported in 4 studies.^8,16,21,22^ Finally, 15 studies earned 18 points or more in the Downs and Black scale and therefore were considered high-quality studies (Supplementary Table 2 online).^8,12,14,17–21,23,26–28,30,31,33^

### Stratified analyses based on type of infection

#### Otitis media

**Fig. 2.**
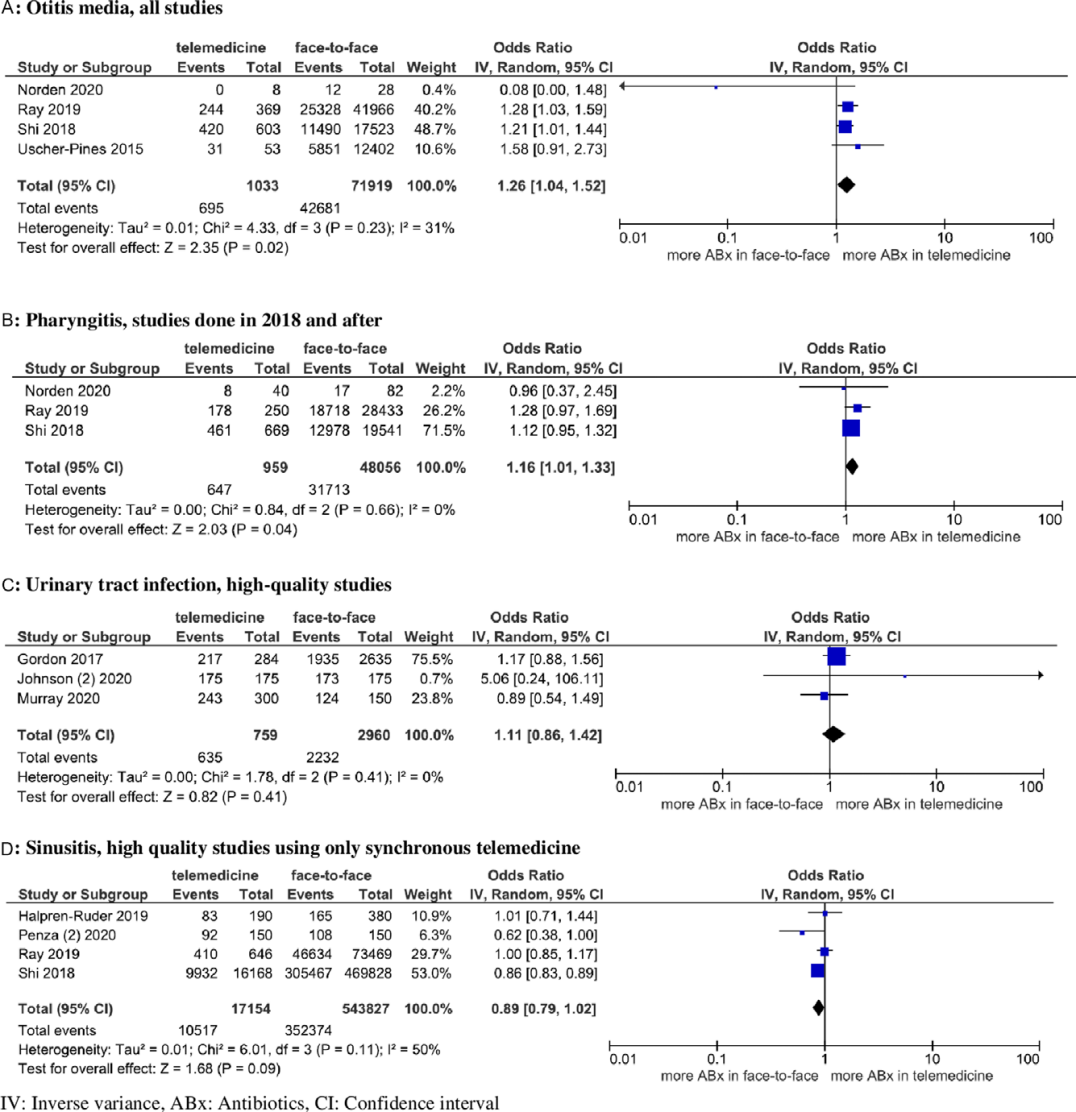
Forest plots for antibiotic prescribing among studies with mild to moderate heterogeneity.

Four retrospective cohort studies evaluated antibiotic prescribing for patients with otitis media.^8,16,21,22^ In total, 1,033 patients (range, 8–603) with otitis media were treated via telemedicine and 71,919 patients (range, 28–41,966) were treated via face-to-face visits. Antibiotic were prescribed for 67.3% of telemedicine and 59.3% of face-to-face visits, respectively. Among these 4 studies, 3 studies^8,21,22^ used claims-based data. Also, 2 studies^8,21^ found more antibiotic prescribing via telemedicine and the other 2 studies^16,22^ did not find a significant difference between the 2 modalities. When those 4 studies were analyzed by meta-analysis, telemedicine use was associated with significantly more antibiotic prescribing compared to face-to-face visits (pooled OR, 1.26; 95% CI, 1.04–1.52) with mild-to-moderate heterogeneity (*P* = .23; I^2^ = 31%) (Fig. 2).

#### Pharyngitis

Five observational studies compared antibiotic prescribing for patients with pharyngitis via telemedicine and face-to-face visits.^8,16,21,22,30^ In total, 1,378 patients (range, 40–669) with pharyngitis were treated via telemedicine and 66,841 patients (range, 82–28,433) were treated via face-to-face visits. Antibiotics were prescribed for 63.4% of telemedicine visits and 61.3% of face-to-face visits. Furthermore, 2 studies^22,30^ found more antibiotic prescribing via telemedicine, and the other 3 studies^8,16,21^ did not find a significant difference between the 2 modalities. Those 5 studies were highly heterogenous (Supplementary Fig. 1 and Supplementary Table 3 online). When limited to more recent studies conducted in 2018 and after, studies were homogenous (*P* = 0.66; I^2^ = 0%), and telemedicine was associated with more antibiotic prescribing compared to face-to-face visits (pooled OR, 1.16; 95% CI, 1.01–1.33) (Fig. 2). In addition to antibiotic prescribing, the utilization of streptococcal testing was evaluated in 2 studies.^8,21^ Although streptococcal testing was ordered in ∼70% of face-to-face visits, it was ordered in only 1%–4% of telemedicine.

#### Urinary tract infection

Four observational studies were included in the meta-analysis for UTI.^7,14,26,30^ In total, 858 patients (range, 98–243) with UTI were treated via telemedicine, and 5,815 patients (range, 150–2,855) were treated via face-to-face visits. Antibiotics were prescribed for 85.4% of telemedicine and 62.6% of face-to-face visits, respectively. Those 4 studies were highly heterogenous (Supplementary Fig. 1 and Supplementary Table 3 online). When the analysis was limited to 3 high-quality studies,^14,26,30^ studies were homogenous (*P* = 0.41; I^2^ = 0%), and there was no significant difference in antibiotic prescribing between telemedicine and face-to-face visits (pooled OR, 1.12; 95% CI, 0.87–1.43) (Fig. 2). In addition to antibiotic prescribing, utilization of urinalysis and urine culture was evaluated in 3 studies.^7,14,26^ Both urinalysis (0%–2.7% in telemedicine and 93%–97.1% in face-to-face visits) and urine culture (0%–7% in telemedicine and 21%–73.1% in face-to-face visits) were utilized less frequently in telemedicine.

#### Sinusitis

Ten observational studies compared antibiotic prescribing for patients with sinusitis via telemedicine and face-to-face visits.^7,8,15,18,21–23,27,30,32^ In total, 21,640 patients (range, 57–16,168) with sinusitis were treated via telemedicine visits and 588,749 patients (range, 100–469,828) were treated via face-to-face visits. Antibiotics were prescribed for 64.8% of telemedicine and 65.9% of face-to-face visits, respectively. The association between antibiotic prescribing and telemedicine compared to face-to-face visits varied among studies. Also, 5 studies^15,18,21,27,32^ reported more antibiotic prescribing in face-to-face visits; 2 studies^7,30^ reported more antibiotic prescribing in telemedicine; and 3 studies^8,22,23^ did not find a statistically significant difference. Those 10 studies were highly heterogenous (Supplementary Fig. 1 and Supplementary Table 3 online). When studies were limited to 4 high-quality studies that used synchronous telemedicine,^8,21,23,27^ studies were still moderately heterogeneous (*P* = 0.11; I^2^ = 50%), and no statistically significant difference in antibiotic prescribing was observed between telemedicine and face-to-face visits (pooled OR, 0.89; 95% CI, 0.79–1.02) (Fig. 2).

#### Upper respiratory infection

Six observational studies evaluated antibiotic prescribing for patients with URIs between telemedicine and face-to-face visits.^8,12,16,21,22,30^ In total, 20,668 patients (range, 132–15,852) with URI were treated via telemedicine and 838,116 patients (range, 85–460,646) were treated via face-to-face visits. Antibiotics were prescribed for 39.9% of telemedicine visits and 29.2% of face-to-face visits, respectively. The association between antibiotic prescribing and telemedicine compared to face-to-face visits varied among studies. In addition, 4 studies^8,12,21,30^ reported more antibiotic prescribing in telemedicine, 1 study^22^ reported more antibiotic prescribing in face-to-face visits, and 1 study^16^ did not find a statistically significant difference. Studies were highly heterogenous, and stratified analyses did not identify any homogenous subgroups (Supplementary Fig. 1 and Supplementary Table 3 online).

### Guideline-concordant antibiotic management

**Fig. 3.**
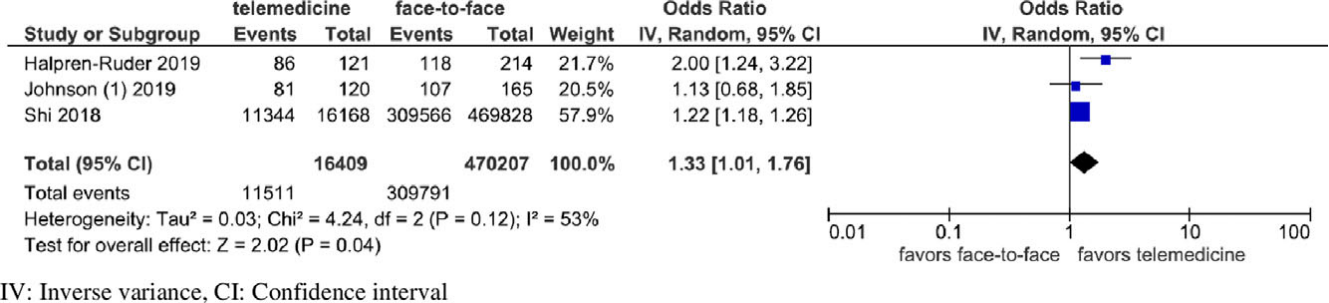
Forest plot for guideline concordant antibiotic management for sinusitis, limited to high-quality studies.

Guideline-concordant antibiotic management for patients with sinusitis was compared between telemedicine and face-to-face visits in 5 studies.^7,8,18,21,23^ Guideline-concordant management was assessed by the choice of guideline-concordant antibiotics in 4 studies,^7,8,18,21^ and antibiotic prescribing only for complicated sinusitis (diagnosed based on history) in 1 study.^23^ Also, 2 studies found more guideline-concordant management in telemedicine,^21,23^ 1 study found more guideline-concordant management in face-to-face visits,^7^ and another 2 studies did not find a significant difference between the 2 modes of delivery.^8,18^ These 5 studies were highly heterogenous (Supplementary Fig. 1 and Supplementary Table 3 online). When the analysis was limited to 3 high-quality studies,^18,21,23^ there was moderate heterogeneity (*P* = .12; I^2^ = 53%), and telemedicine use was associated with significantly more guideline-concordant management (pooled OR, 1.33; 95% CI, 1.01–1.76) (Fig. 3). Guideline-concordant management for other diagnoses was not investigated due to a small number of studies.

### Publication bias

We assessed publication bias by creating funnel plots for studies evaluating each diagnosis (sinusitis, URI, UTI, pharyngitis, and OM) (Supplementary Fig. 2). Aside from studies with extreme odds ratios (<0.2 or >5), studies were reasonably balanced around the pooled odds ratios, and studies with null results were included. Thus, there was little evidence of publication bias.

## Discussion

In this systematic literature review and meta-analysis, antibiotics were more frequently prescribed via telemedicine compared to face-to-face visits for patients with otitis media and pharyngitis. Telemedicine was associated with more guideline-concordant management for patients with sinusitis. Nevertheless, the overall interpretation of those results requires caution because there was substantial heterogeneity among studies.

The decision to prescribe antibiotics is a complex process involving provider factors, patient factors, and external factors.^34^ Outpatient providers’ antibiotic prescribing can be driven by the provider’s anxiety or fear regarding diagnostic uncertainty, complications from an infection, and lack of continuity of care. Additionally, outpatient providers may try to maintain good relationships with patients and increase patient satisfaction by prescribing antibiotics.^35^ It is also suggested that outpatient providers may feel that antimicrobial resistance is related to transmission in hospital settings and is not driven by outpatient antibiotic prescribing.^36^ Additionally, patients’ demand for antibiotics could pressure a provider to prescribe an antibiotic.^37^ Outpatient antibiotic overprescribing is probably a result of tightly interacting provider and patient factors, as well as external factors such as organizational pressures for time and financial incentives.^34^ Through telemedicine, some of these factors may be stronger and others may be weaker. For example, a thorough physical examination is lacking with telemedicine. Also, there would be higher thresholds for ordering lab tests or imaging during telemedicine visits compared to face-to-face visits. The lack of physical examination and diagnostic modalities may increase providers’ anxiety about diagnostic uncertainty; therefore, antibiotics may be prescribed more often. In contrast, patients’ demand for antibiotics and providers’ perception for that might be weaker via telemedicine, especially in the case of asynchronous telemedicine.

In our study, telemedicine visits for pharyngitis and otitis media were associated with higher rates of antibiotic prescribing. One possible explanation for this is the lack of physical examination in telemedicine, which is necessary to make a correct diagnosis for streptococcal pharyngitis.^38^ The availability of streptococcal rapid testing was lower via telemedicine. Providers may feel that it is easier to prescribe antibiotics to treat pharyngitis, rather than pursuing a time-consuming process to obtain reliable physical examination or rapid testing to make a correct diagnosis. Similar to the diagnosis of streptococcal pharyngitis, the diagnosis of otitis media requires an otoscopic examination.^39^ Although new technologies, such as digital videoscopes and smart phones, may enable remote ear and oropharyngeal examinations, they are not yet routinely available in primary care settings.^40^ Thus, it is possible that otitis media is overdiagnosed in telemedicine settings given the lack of otoscopic examination, and this could be driving increased antibiotic prescribing. Improvements in remote otoscopic examination may eliminate this barrier, but that would require further study.

Contrary to pharyngitis and otitis media, we did not observe a significant difference in antibiotic prescribing for patients with sinusitis or URI when care was delivered during telemedicine or face-to-face visits. For the management of sinusitis, antibiotics are only indicated in cases of severe disease, worsening course, or persistent illness, which can be differentiated with clinical history alone without the need for physical examination or diagnostic tests.^41^ Therefore, diagnostic uncertainty may not be greatly different for sinusitis between the 2 modes of delivery. Interestingly, telemedicine was associated with more guideline-concordant management for patients with sinusitis. Although it is possible that there was less patient demand for antibiotic prescribing in telemedicine, the true reason for that observation remains unclear. Diagnosis of URI is ultimately made after excluding other diagnoses that mimic URI. Patients with URI are probably a more heterogeneous group than those with other diagnoses, and it is difficult to make conclusions about antibiotic prescribing for URI with the heterogeneity of the included studies. A significant proportion (30%–40%) of patients with URI received antibiotics even though antibiotics are almost never indicated for URI, indicating room for improvement in future ASP activities.

We did not detect a significant difference in antibiotic prescribing for patients with UTI in our meta-analysis. UTI is a diagnosis for which treatment with antibiotics is almost always indicated.^42^ Therefore, it is not surprising that there was not a significant difference in antibiotic prescribing between telemedicine and face-to-face visits. On the other hand, the appropriateness of treatment may be affected by the mode of care delivery because significantly fewer urinalyses and urine cultures were ordered during telemedicine visits. Interestingly, 2 studies that evaluated either first-line antibiotics or guideline-recommended antibiotics showed that telemedicine provided more appropriate treatment.^7,14^ Moreover, studies that investigated revisit as a marker for treatment failure did not report an increase in revisiting after a telemedicine encounter.^14,26^ Although it is possible that telemedicine can provide similarly effective but lower-cost care for UTI, this hypothesis will need to be validated by future studies.

Our systematic literature review and meta-analysis have several limitations. First, due to the heterogeneity among studies, our findings should be interpreted with caution. The studies varied in the study settings, population, and type of telemedicine and face-to-face visits. Due to the heterogeneity among studies, we elected not to perform a meta-analysis including all diagnoses but rather to conduct meta-analyses for each diagnosis. We also tried to determine the sources of heterogeneity by conducting several stratified analyses, but we could not conduct some of the stratified analyses due to the limited number of studies. Therefore, it is possible that there remained substantial residual heterogeneity. Although we acknowledge this limitation, we believe our findings will provide very important preliminary information for future studies. Second, most of the included studies used administrative codes to identify infections without confirmation by chart review. Administrative codes are not always accurate, but the reported positive predictive values for common infection such as pharyngitis or bronchitis were fairly good, ∼80%.^43^ Third, there may be some bias due to lack of information for inclusion in meta-analysis. We asked the corresponding authors of 6 studies to provide additional information, but we could include only 1 study with additional information. Fourth, many of the included studies had significant imbalance in sample size between telemedicine and face-to-face visits. Unmeasured biases may have affected the selection of patients seen via telemedicine. Finally, we did not fully investigate the appropriateness of treatment and follow-up of care, which are also very important components in assessing care variation between telemedicine and face-to-face visits. It is challenging to assess appropriateness of antibiotic prescribing using a retrospective study design without extensive chart review.

The use of telemedicine may change after the COVID-19 pandemic has been controlled, but the adaptation trajectory of these technologies has been forever changed.^44^ As we expect continued high-volume use of telemedicine in outpatient settings, it would be very important to correctly understand how telemedicine affects antibiotic prescribing. To validate or further investigate our preliminary findings, large-scale, well-designed studies with comprehensive assessments of antibiotic prescribing for common outpatient infections will be warranted.

In conclusion, our systematic review and meta-analysis found that telemedicine visits were associated with higher rates of antibiotic prescribing in some diagnoses, such as otitis media and pharyngitis. It seemed that providers overprescribed antibiotics for patients with diagnoses where antibiotics were not always indicated via for both modes of delivery. The interpretation of these findings requires caution due to substantial heterogeneity among available studies. Large-scale, well-designed studies with comprehensive assessment of antibiotic prescribing for common outpatient infections comparing telemedicine and face-to-face visits are needed to validate our findings.

## Supplementary material

For supplementary material accompanying this paper visit https://doi.org/10.1017/ash.2021.179. click here to view supplementary material
