# Supplementary material for: Outpatient antibiotic prescribing for common infections via telemedicine versus face-to-face visits: Systematic literature review and meta-analysis
Source: Antimicrob Steward Healthc Epidemiol. 2021 Aug 31;1(1):e24. doi: 10.1017/ash.2021.179 (PMC9495625; doi:10.1017/ash.2021.179)
Supplement: Supplementary file 1 [file S2732494X21001790sup001.docx]

Supplemental Table 1. Search strategies

**PubMed**

"Office Visits"[Mesh] OR Urgent care[Text Word] OR Office visit[Text Word] OR Outpatient visits[Text Word] OR Primary care visit[Text Word] OR primary care visits[Text Word] OR PCP visit[Text Word] OR PCP visits[Text Word] OR In person[Text Word] OR Face to face[Text Word] OR F2F[Text Word] OR Traditional care[Text Word] OR Traditional visit [Text Word] OR traditional visits[Text Word] OR Standard care[Text Word] OR "Ambulatory Care"[Mesh] OR "Primary Health Care"[Mesh]

AND

antibiotic[Text Word] OR antibiotics[Text Word] OR antibacterial[Text Word] OR antibacterials[Text Word] OR antimicrobial[Text Word] OR antimicrobials[Text Word] OR Anti microbial[Text Word] OR anti microbials[Text Word] OR Anti bacterial[Text Word] OR anti bacterials[Text Word] OR Prescribing practice[Text Word] OR prescribing practices[Text Word] OR antiinfective[Text Word] OR antiinfectives[Text Word] OR Anti infective[Text Word] OR anti infectives[Text Word] OR "Inappropriate Prescribing"[Mesh] OR "Anti-Bacterial Agents"[Mesh]

OR "Practice Patterns, Physicians'"[Mesh] OR "Drug Prescriptions"[Mesh] OR "Sinusitis/drug therapy"[Mesh] OR "Urinary Tract Infections/drug therapy"[Mesh] OR "Respiratory Tract Infections/drug therapy"[Mesh] OR "Antimicrobial Stewardship"[Mesh] OR "Drug Utilization" [Mesh]

AND

"Videoconferencing"[Mesh] OR “Text Messaging”[Mesh] OR "Electronic Mail"[Mesh] OR "Wireless Technology"[Mesh] OR "Telecommunications"[Mesh:NoExp] OR "Telephone"[Mesh] OR "Telemedicine"[Mesh] OR "Internet"[Mesh] OR Evisit[Text Word] OR evisits[Text Word] OR Telemedicine[Text Word] OR Video consultation[Text Word] OR Teleconferencing[Text Word] OR Mobile health[Text Word] OR Telehealth[Text Word] OR Ehealth[Text Word] OR Mhealth[Text Word] OR “E health”[Text Word] OR “M health” [Text Word] OR Mobile application[Text Word] OR mobile applications[Text Word] OR Smartphone[Text Word] OR smartphones [Text Word] OR Teleconsultation[Text Word] OR Remote[Text Word] OR Teleconferencing[Text Word] OR Teleconsultation[Text Word] OR virtual [Text Word] OR Email[Text Word] OR Electronic mail[Text Word] OR E mail[Text Word] OR SMS[Text Word] OR Short message service[Text Word] OR Wireless technology[Text Word] OR Wireless technologies[Text Word] OR Telephone[Text Word] OR Phone[Text Word] OR phones [Text Word] OR Telecommunication[Text Word] OR Video conferencing[Text Word] OR Videoconferencing[Text Word] OR Text messaging[Text Word] OR Texting[Text Word] OR Text message[Text Word] OR text messages [Text Word]

= 930

**CINAHL**

#1

(MH "Office Visits") OR (MH "Ambulatory Care") OR (MH "Ambulatory Care Facilities+") OR (MH "Primary Health Care")

OR

"Urgent care" OR "Office visit*" OR "Outpatient visit*" OR "Primary care visit*" OR "PCP visit*" OR "In person" OR "Face to face" OR F2F OR "Traditional care" OR "Traditional visit*" OR "Standard care"

#2

(MH "Inappropriate Prescribing") OR (MH "Antibiotics+") OR (MH "Prescriptions, Drug+") OR (MH "Sinusitis+/TH") OR (MH "Urinary Tract Infections+/TH") OR (MH "Respiratory Tract Infections+/TH") OR (MH "Antimicrobial Stewardship") OR (MH "Drug Utilization+") OR (MH "Practice Patterns")

OR

antibiotic OR antibiotics OR antibacterial OR antibacterials OR antimicrobial OR antimicrobials OR "Anti microbial*" OR "Anti bacterial*" OR "Prescribing practice*" OR antiinfective OR antiinfectives OR "Anti infective*"

#3

(MH "Telemedicine+") OR (MH "Internet") OR (MH "Email") OR (MH "Videoconferencing+") OR (MH "Wireless Communications") OR (MH "Telecommunications") OR (MH "Telehealth+") OR (MH "Telephone+") OR (MH "Text Messaging+") OR (MH "Teleconferencing") OR (MH "User-Computer Interface+")

OR

Evisit* OR telemedicine OR "Video consultation*" OR "Mobile health" OR Telehealth OR Ehealth OR Mhealth OR "E health" OR "M health" OR "Mobile application*" OR Smartphone* OR Teleconsultation* OR Remote OR Teleconferencing OR Teleconsultation* OR virtual OR Email OR "Electronic mail" OR "E mail" OR SMS OR "Short message service*" OR "Wireless technolog*" OR Telephone OR Phone* OR Telecommunication* OR "Video conferencing" OR Videoconferencing OR "Text messag*" OR Texting

#1 AND #2 AND #3 = 566

**Embase**

#1

'ambulatory care'/exp OR 'primary health care'/exp OR 'outpatient department'/exp OR ('urgent care' OR 'office visit*' OR 'outpatient visit*' OR 'primary care visit*’ OR 'pcp visit*' OR 'in person' OR 'face to face' OR f2f OR 'traditional care' OR 'traditional visit*' OR 'standard care'):ab,ti

#2

'telemedicine'/exp OR 'telehealth'/exp OR 'computer interface'/exp OR 'telephone'/exp OR 'mobile phone'/exp OR 'teleconference'/exp OR 'e-mail'/exp OR 'internet'/exp OR 'videoconferencing'/exp OR 'text messaging'/exp OR (evisit* OR telemedicine OR 'video consultation*' OR 'mobile health' OR telehealth OR ehealth OR mhealth OR 'e health' OR 'm health' OR 'mobile application*' OR smartphone* OR 'remote visit*' OR 'remote consultation*' OR teleconferencing OR teleconsultation* OR 'virtual visit*' OR 'virtual care' OR email OR 'electronic mail' OR 'e mail' OR sms OR 'short message service*' OR 'wireless technolog*'OR telephone OR phone OR telecommunication* OR 'video conferencing' OR videoconferencing OR 'text messag*' OR texting):ab,ti

#3

'respiratory tract infection'/exp AND ('disease management'/lnk OR 'drug therapy'/lnk)

OR

'sinusitis'/exp AND ('disease management'/lnk OR 'drug therapy'/lnk)

OR

'respiratory tract infections'/exp AND ('disease management'/lnk OR 'drug therapy'/lnk)

OR

'antiinfective agent'/exp OR 'electronic prescribing'/exp OR 'inappropriate prescribing'/exp OR 'antimicrobial stewardship'/exp OR 'drug utilization'/exp

OR

(antibiotic OR antibiotics OR antibacterial OR antibacterials OR antimicrobial OR antimicrobials OR 'anti microbial*' OR 'anti bacterial*' OR antiinfective OR antiinfectives OR 'anti infective*'):ab,ti

#1 AND #2 AND #3 = 1643

**Cochrane CENTRAL**


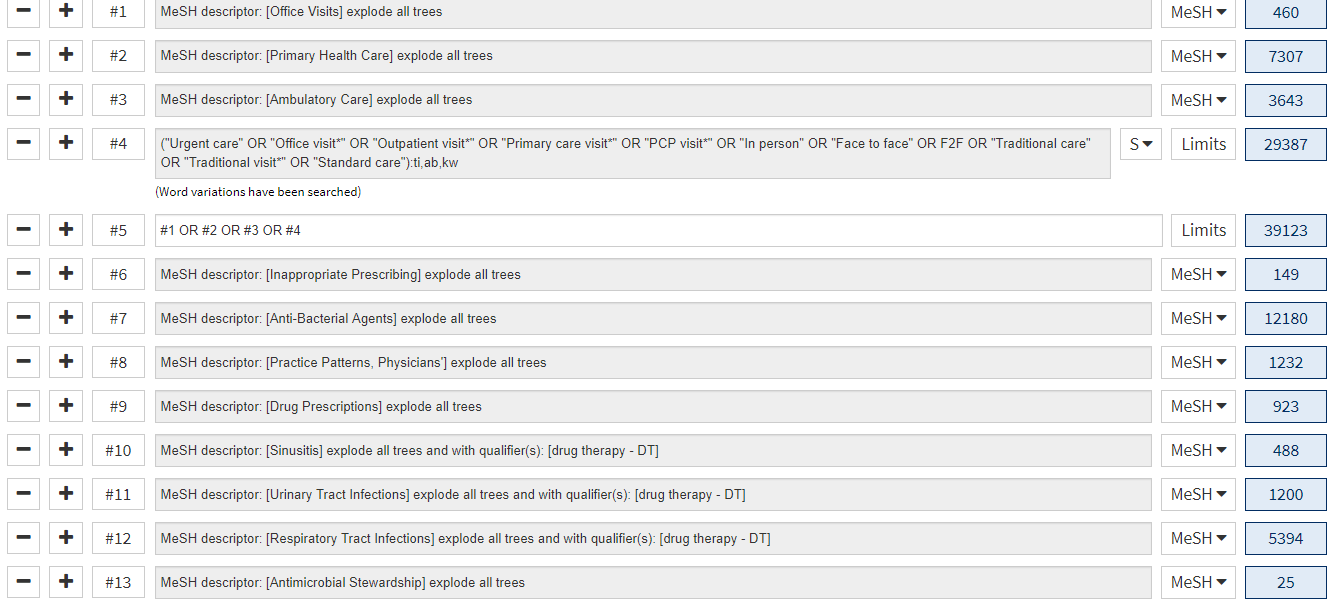


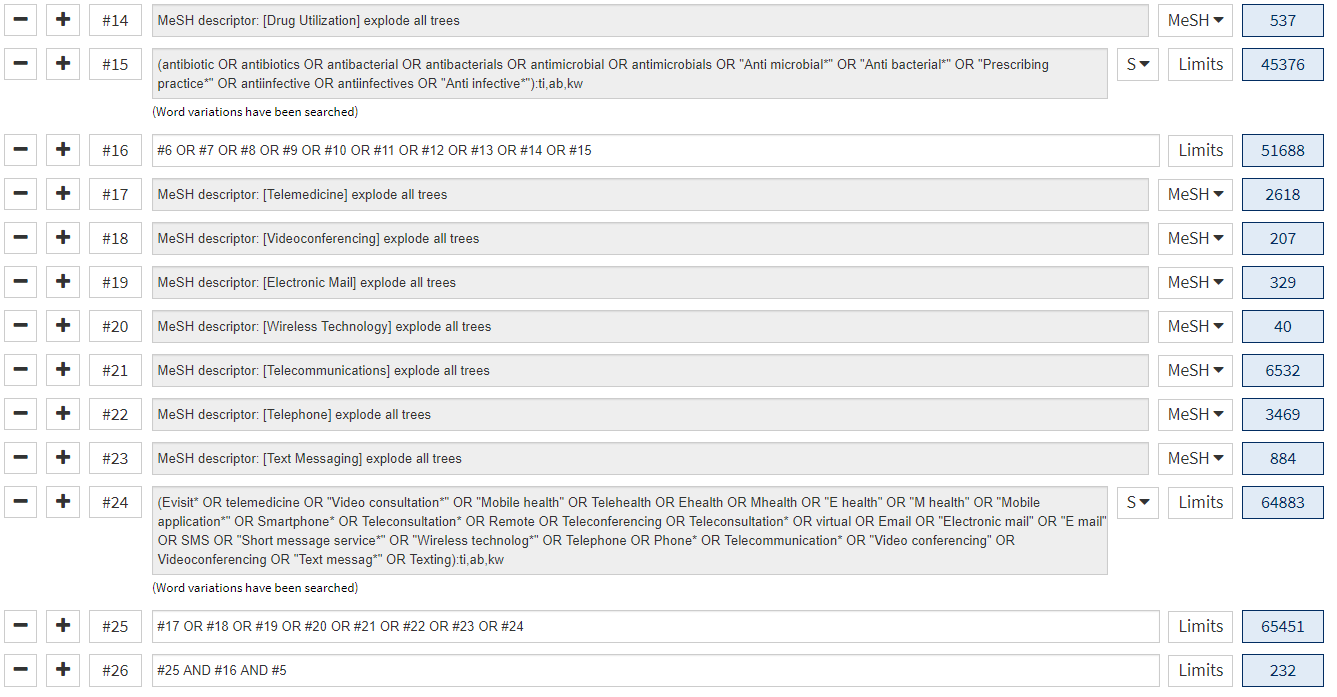


= 220 when limited to trials

Supplemental Table 2. Details of Downs and Black scale for each study

| First author/ publication year/location | Reporting (Max 11 points) | External validity (Max 3 points) | Internal validity – bias (Max 7 points) | Internal validity – confounding (selection bias) (Max 6 points) | Power (Max 1 point) | Total score |
| --- | --- | --- | --- | --- | --- | --- |
| Bruxvoort/2020/ California, US [19] | 9 | 3 | 5 | 2 | 0 | 19 |
| Davis/2018/ Colorado, US [15] | 7 | 2 | 5 | 3 | 0 | 17 |
| Ewen/2015/ Delaware, US [20] | 8 | 3 | 5 | 4 | 0 | 20 |
| Gordon/2017/ US [30] | 9 | 3 | 5 | 4 | 0 | 21 |
| Halpren-Ruder/2019/ Pennsylvania, US [23] | 8 | 3 | 5 | 3 | 0 | 19 |
| Hersh/2019/ Utah, US [25] | 4 | 3 | 3 | 3 | 0 | 13 |
| Huibers/2014 Central Denmark [24] | 7 | 3 | 4 | 3 | 0 | 17 |
| Johnson (1)/2019/ Michigan, US [18] | 10 | 3 | 5 | 4 | 1 | 23 |
| Johnson (2)/2020/ Michigan, US [14] | 10 | 3 | 5 | 4 | 1 | 23 |
| Lovell/2019/ Utah, US [31] | 9 | 3 | 5 | 4 | 0 | 21 |
| McKinstry/2002/ West Lothian, UK [33] | 8 | 3 | 5 | 5 | 1 | 22 |
| Mehrotra/2013/ Pennsylvania, US [7] | 5 | 3 | 5 | 3 | 0 | 16 |
| Miller/2020/ Massachusetts, US [32] | 8 | 3 | 5 | 1 | 0 | 17 |
| Murray/2020/ Minnesota, US [26] | 7 | 3 | 5 | 3 | 0 | 18 |
| Norden/2020/ California, US [16] | 6 | 3 | 5 | 3 | 0 | 17 |
| Penza (1)/2020/ Minnesota, US [28] | 8 | 3 | 5 | 3 | 0 | 19 |
| Penza (2)/2020/ Minnesota, US [27] | 7 | 3 | 5 | 3 | 0 | 18 |
| Ray/2019/ US [8] | 8 | 3 | 5 | 4 | 0 | 20 |
| Schmidt/2017/ North Carolina, US [29] | 5 | 3 | 5 | 3 | 0 | 16 |
| Shi/2018/ US [21] | 7 | 3 | 5 | 4 | 0 | 19 |
| Tan/2016/ Nevada, US [12] | 9 | 3 | 5 | 3 | 0 | 20 |
| Uscher-Pines/2015/ California, US [22] | 6 | 2 | 5 | 4 | 0 | 17 |
| Yao/2019/ New York, US [17] | 7 | 3 | 5 | 3 | 0 | 18 |

Supplemental table 3. Results of stratified analyses

| Diagnosis | Analysis | N of studies | Pooled OR (95%CI) | P value | Heterogeneity | |
| --- | --- | --- | --- | --- | --- | --- |
|  |  |  |  |  | I^2^ (%) | P value |
| Otitis media | All studies | 4 | 1.26 (1.04-1.52) | 0.02* | 31 | 0.23 |
|  | Studies in 2018-2020 | 3 | 1.22 (0.98-1.52) | 0.08 | 44 | 0.17 |
|  | Studies using claim-based data | 3 | 1.25 (1.10-1.43) | <0.001* | 0 | 0.63 |
| Pharyngitis | All studies | 5 | 1.58 (1.08-2.31) | 0.02* | 85 | <0.001 |
|  | High-quality studies | 3 | 1.51 (0.97-2.36) | 0.07 | 89 | <0.001 |
|  | Only adults | 3 | 1.42 (0.80-2.54) | 0.23 | 84 | 0.002 |
|  | Only clinic visit as face-to-face visits | 3 | 1.47 (0.97-2.24) | 0.07 | 87 | <0.001 |
|  | Only urgent care as face-to-face visits | 4 | 1.45 (1.04-2.02) | 0.03* | 73 | 0.01 |
|  | Urgent care & high-quality studies | 3 | 1.51 (1.05-2.18) | 0.03* | 81 | 0.005 |
|  | Studies in 2018-2020 | 3 | 1.16 (1.01-1.33) | 0.04* | 0 | 0.66 |
| UTI | All studies | 4 | 2.57 (0.88-7.46) | 0.08 | 86 | <0.001 |
|  | High-quality studies | 3 | 1.11 (0.86-1.42) | 0.41 | 0 | 0.41 |
| Sinusitis | All studies | 10 | 0.86 (0.70-1.06) | 0.16 | 91 | <0.001 |
|  | Synchronous telemedicine use | 6 | 0.88 (0.73-1.07) | 0.21 | 78 | <0.001 |
|  | High-quality studies | 6 | 0.80 (0.60-1.05) | 0.11 | 93 | <0.001 |
|  | Only adults | 6 | 0.67 (0.52-0.86) | 0.002* | 89 | <0.001 |
|  | Synchronous telemedicine & high-quality studies | 4 | 0.89 (0.79-1.02) | 0.09 | 50 | 0.11 |
|  | Adults & high-quality studies | 3 | 0.44 (0.18-1.06) | 0.07 | 93 | <0.001 |
|  | Adults & synchronous telemedicine | 4 | 0.74 (0.52-1.07) | 0.12 | 84 | <0.001 |
|  | Only clinic visit as face-to-face visits | 6 | 1.00 (0.86-1.18) | 0.96 | 85 | <0.001 |
|  | Only urgent care as face-to-face visits | 5 | 0.79 (0.57-1.09) | 0.16 | 93 | <0.001 |
|  | Adults & clinic visits | 3 | 0.90 (0.77-1.04) | 0.14 | 78 | 0.01 |
|  | Clinic visits & high-quality studies | 3 | 0.96 (0.84-1.09) | 0.53 | 70 | 0.04 |
|  | Urgent care & high-quality studies | 4 | 0.90 (0.66-1.24) | 0.54 | 94 | <0.001 |
|  | Asynchronous telemedicine use | 3 | 0.76 (0.12-4.90) | 0.77 | 95 | <0.001 |
|  | Studies in 2018-2020 | 7 | 0.71 (0.57-0.87) | <0.001* | 87 | <0.001 |
|  | Adults & studies in 2018-2020 | 5 | 0.57 (0.42-0.76) | <0.001* | 90 | <0.001 |
|  | High-quality studies in 2018-2020 | 5 | 0.71 (0.53-0.94) | 0.02 | 89 | <0.001 |
| URI | All studies | 6 | 1.18 (0.59-2.39) | 0.64 | 100 | <0.001 |
|  | High-quality studies | 4 | 1.67 (0.72-3.89) | 0.23 | 100 | <0.001 |
|  | Only clinic visit as face-to-face visits | 4 | 1.16 (0.46-2.94) | 0.75 | 100 | <0.001 |
|  | Only urgent care as face-to-face visits | 5 | 1.18 (0.78-1.78) | 0.43 | 99 | <0.001 |
|  | Urgent care & high-quality studies | 3 | 1.31 (0.76-2.27) | 0.33 | 99 | <0.001 |
|  | Studies in 2018-2020 | 3 | 1.65 (0.55-4.94) | 0.37 | 100 | <0.001 |
| Sinusitis, guideline-concordance | All studies | 5 | 1.10 (0.87-1.39) | 0.43 | 86 | <0.001 |
|  | Studies in 2018-2020 | 4 | 1.20 (1.00-1.44) | 0.05* | 65 | 0.04 |
|  | High-quality studies | 3 | 1.33 (1.01-1.76) | 0.04 | 53 | 0.12 |

UTI: Urinary tract infection, URI: Upper respiratory infection

Supplemental Figure 1. Forest plots for selected diagnoses

**A: Pharyngitis, all studies**

**B: Urinary tract infection, all studies**

**C: Sinusitis, all studies**

**D: Upper respiratory infection, all studies**

**E: Sinusitis, all studies evaluating guideline-concordance**

ABX: Antibiotics

Supplemental Figure 2. Funnel plots for each diagnosis

A: Funnel plot for sinusitis

B: Funnel plot for upper respiratory infection (URI)

C: Funnel plot for urinary tract infection (UTI)

D: Funnel plot for Pharyngitis


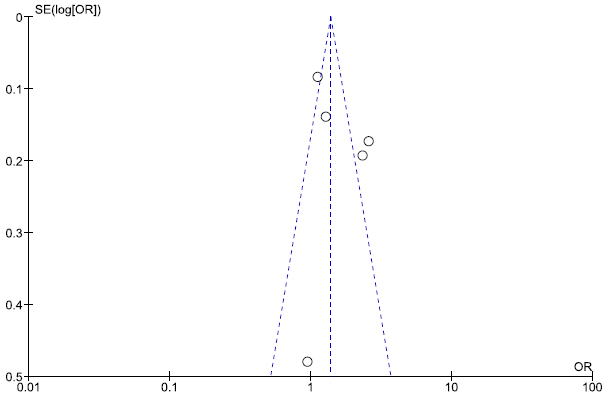


E: Funnel plot for otitis media

SE: Standard error, OR: Odds ratio
